# Oral administration of cytosolic PLA2 inhibitor arachidonyl trifluoromethyl ketone ameliorates cauda equina compression injury in rats

**DOI:** 10.1186/s12974-015-0311-y

**Published:** 2015-05-15

**Authors:** Mushfiquddin Khan, Anandakumar Shunmugavel, Tajinder S Dhammu, Fumiyo Matsuda, Avtar K Singh, Inderjit Singh

**Affiliations:** Department of Pediatrics, Medical University of South Carolina, Charleston, SC 29425 USA; School of Health Science, Kagoshima University, Kagoshima, Japan; Department of Pathology and Laboratory Medicine, Medical University of South Carolina, Charleston, SC USA; Ralph H. Johnson VA Medical Center, Charleston, SC USA

**Keywords:** Lumbar spinal canal stenosis, Cauda equina compression, Cytosolic phospholipase A2, Neuroinflammation, Arachidonyl trifluoromethyl ketone, Pain, Motor function

## Abstract

**Background:**

Phospholipase A2 (PLA2)-derived proinflammatory lipid mediators such as prostaglandin E2 (PGE2), leukotrienes B4 (LTB4), lysophosphatidylcholine (LPC), and free fatty acids (FFA) are implicated in spinal cord injury (SCI) pathologies. Reducing the levels of these injurious bioactive lipid mediators is reported to ameliorate SCI. However, the specific role of the group IVA isoform of PLA2 cytosolic PLA2 (cPLA2) in lumbar spinal canal stenosis (LSS) due to cauda equina compression (CEC) injury is not clear. In this study, we investigated the role of cPLA2 in a rat model of CEC using a non-toxic cPLA2-preferential inhibitor, arachidonyl trifluoromethyl ketone (ATK).

**Methods:**

LSS was induced in adult female rats by CEC procedure using silicone blocks within the epidural spaces of L4 to L6 vertebrae. cPLA2 inhibitor ATK (7.5 mg/kg) was administered by oral gavage at 2 h following the CEC. cPLA2-derived injurious lipid mediators and the expression/activity of cPLA2, 5-lipoxygenase (5-LOX), and cyclooxygenase-2 (COX-2) were assessed. ATK-treated (CEC + ATK) were compared with vehicle-treated (CEC + VEH) animals in terms of myelin levels, pain threshold, and motor function.

**Results:**

ATK treatment of CEC animals reduced the phosphorylation of cPLA2 (pcPLA2) determined by Western blot, improved locomotor function evaluated by rotarod task, and reduced pain threshold evaluated by mechanical hyperalgesia method. Levels of FFA and LPC, along with PGE2 and LTB4, were reduced in CEC + ATK compared with CEC + VEH group. However, ATK treatment reduced neither the activity/expression of 5-LOX nor the expression of COX-2 in CEC + VEH animals. Increased cPLA2 activity in the spinal cord from CEC + VEH animals correlated well with decreased spinal cord as well as cauda equina fiber myelin levels, which were restored after ATK treatment.

**Conclusion:**

The data indicate that cPLA2 activity plays a significant role in tissue injury and pain after LSS. Reducing the levels of proinflammatory and tissue damaging eicosanoids and the deleterious lipid mediator LPC shows therapeutic potential. ATK inhibits cPLA2 activity, thereby decreasing the levels of injurious lipid mediators, reducing pain, improving functional deficits, and conferring protection against LSS injury. Thus, it shows potential for preclinical evaluation in LSS.

## Background

Lumbar spinal canal stenosis (LSS) is a stable disorder with mild to severe disability, degeneration, and pain. Low back pain (LBP) due to LSS is common, costly, mechanistically complex, and clinically challenging [1]. It is the most frequent neurologic reason that the elderly undergo spinal surgery. While the benefit of surgery in older people is limited in terms of both effectiveness and recovery time [2], nonsurgical options for LSS management are even less satisfactory [3]. Chronic LSS leads to the compression of cauda equina (CE) fibers [4]. Sensitization of CNS and the peripheral nervous system results in neuropathic syndrome [5], a common and severely debilitating condition affecting millions of people worldwide [6]. The pathological manifestation of chronic cauda equina compression (CEC) is intermittent neurogenic claudication/pseudoclaudication. Neurogenic claudication consists of pain in the buttocks or legs. Lipotoxicity, inflammation, and cell loss are involved in neuropathic pain and functional deficits. Activation of cytosolic PLA2 (cPLA2) has been shown to produce lipoxidative toxicity, leading to inflammation and pain [7].

Phospholipase A2s (PLA2s) hydrolyze a fatty acid from membrane phospholipids’ *sn*-2 position, a site enriched with polyunsaturated fatty acids. To date in the mammalian system, more than two dozen identified isoforms of PLA2 can be classified into three major categories: Ca^2+^-dependent secretory PLA2 (sPLA2), Ca^2+^-dependent cytosolic PLA2 (cPLA2), and Ca^2+^-independent PLA2 (iPLA2) [8]. Of these, group via cPLA2 is the most important PLA2 isozyme because it has been implicated in the release of arachidonic acid (AA). cPLA2 activity is increased due to its phosphorylation on Ser^505^ by MAP kinase as reviewed [9]. Phosphorylation of Ser^505^ is important in the activation of cPLA2 *in vivo* because overexpression of mutant Ser^505^ fails to enhance AA release as seen with normal Ser^505^ [10,11]. Activation of MAP kinase has also been shown to participate in hypersensitivity after nerve injury [12]. In a contusive spinal cord injury (SCI) at the T10 levels, cPLA2 is also activated by ERK1/2 [13].

AA is as an important intracellular signaling molecule [14] and serves as a precursor of eicosanoids that are pleiotropic bioactive lipid mediators [15]. AA is metabolized primarily by two different groups of enzymes, prostaglandin synthases (cyclooxygenases (COX)), and lipoxygenases (LOX). Metabolic products include prostaglandins, thromboxanes, hydroxyeicosatetraenoic acids, and leukotrienes. cPLA2 is considered significant for reasons that include its preference for AA-containing phospholipids and the fact that physiological increases in cytosolic-free Ca^2+^ cause translocation of this PLA2 to the membrane compartments (in particular the nuclear envelope, endoplasmic reticulum, and Golgi body) [16], where COX and LOX also preferentially localize [17,18]. AA-containing phosphatidylcholine (PC) is the most preferred substrate of cPLA2, giving rise to lysophosphatidylcholine (LPC) and free AA [7,8,19-21]. A significant increase in cPLA2 activity and AA levels following contusive spinal cord injury at T9 to T10 levels has been reported [22,23]. AA-derived prostaglandins and leukotrienes are associated with oxidative damage in the contusive spinal cord injury [24]. Prostaglandin E2 (PGE2) can increase local blood flow and leukocyte infiltration, enhancing vascular permeability and cytokine production [25]. This bioactive lipid is also involved in CEC-induced spinal inflammation and pain [26].

The mechanisms underlying neuropathic pain are complex and multifactorial [27]. Axonal degeneration following nerve injury causes neuropathic pain [28]. Demyelination is also a characteristic of cPLA2-mediated spinal cord injury [13]. In the AA cascade, bioactive substances are released in response to the mechanical compression of the cauda equina and nerve root, inducing hypersensitivity to neuropathic pain [29]. SCI-associated secondary damage is also characterized by the induction of COX-2 [30]. Activation of PGE2 receptors leads to Ca^2+^ dysregulation, facilitating cPLA2 activation and potentiating the release of AA metabolites [31]. Spinal prostaglandins are directly involved in mediating allodynia and inflammation after spinal cord injury [32]. Proinflammatory and nociceptive stimuli can also induce inducible nitric oxide synthase (iNOS), which produces a large amount of nitric oxide (^•^NO) for a sustained period of time [33]. In turn, iNOS modulates cPLA2-dependent AA release and PGE2 formation [34]. Induction of iNOS mainly results in the formation of peroxynitrite (ONOO^−^), a product of an instantaneous reaction between ^•^NO and superoxide. ONOO^−^ has been implicated in neuropathic pain following nerve injury [35]. A positive correlation exists between NOS activity and both PGE2 generation and the upregulation of cPLA2 and COX-2 [36]. COX-2 is reported to increase inflammation in the spinal cord and subsequent PGE2 levels in cerebrospinal fluid [37]. Hence, a cPLA2 inhibition strategy is anticipated to reduce COX-2-derived inflammatory and nociceptive mediators, thus mitigating pain and spinal tissue degeneration.

We hypothesize that the cPLA2 inhibitor arachidonyl trifluoromethyl ketone (ATK) will reduce inflammation and pain, leading to functional recovery following CEC-induced LSS. ATK is an analog of AA in which the carboxyl group is replaced by a trifluoromethyl ketone group [38]. ATK inhibits cPLA2 both *in vitro* and *in vivo* [39-42]. It is a potent and selective slow binding inhibitor of the 85 kDa cPLA2 (group IVA cPLA2α). ATK inhibits the activity cPLA2 without affecting the 14 kDa sPLA2 and minimally affecting iPLA2. The efficacy of ATK has been reported in animal models of spinal cord injury [13,24] and other neurodegenerative diseases [43].

In the present study, we observed that an oral administration of ATK of CEC female rats decreased the phosphorylation/activation of cPLA2, resulting in reduced levels of proinflammatory bioactive injurious lipids, including PGE2, leukotriene B4 (LTB4), free fatty acids (FFA), and LPC. The ATK treatment also blocked demyelination, reduced the threshold of pain, and improved coordination and balance in a 2-week LSS study.

## Materials and methods

### Animals

Adult female Sprague–Dawley rats (approximately 300 g) from Harlan laboratory (Indianapolis, IN, USA) were used in this study. Animals were housed in a 12:12-h light:dark cycle with free access to food and water. All animal experiments were carried out under the control of the Institutional Animal Care and Use Committee (IACUC) in accordance with guidelines for animal experiments of the Medical University of South Carolina.

### Surgical procedure and drug administration

LSS was induced by following the CEC method of Watanabe *et al*. [44]. Animals were anesthetized by intraperitoneal injection of ketamine-xylazine cocktail (80 and 10 mg/kg body weight, respectively). After confirming the validity of anesthesia by toe pinching, the animals were depilated on the dorsal spine line and placed in a prone position. An incision was made to the middle of the spine at the L4 to S2 level to reveal the L4 to L5 lamina through Leica surgical microscope (Leica Microsystems, Buffalo Grove, IL, USA). Paraspinal muscles were separated from the spinal processes at the L4 to L6 level to expose the ligamentum flavum. A piece of silicone block (length: 4 mm, width: 1 mm, thickness: 1 mm; Bentec Medical Inc, Woodland, CA, USA) was placed into the epidural space under the L4 to L5 vertebrae. In the same way, another silicon block was placed under the L5 to L6 vertebrae. Proper positioning of the silicon blocks was confirmed by MRI scan. After surgery, the wound was irrigated with PBS. Muscle layers and skin were sutured with polysorb 4 (Medline Industries, Inc., Mundelein, IL, USA). Sham-operated animals underwent all the surgical procedures except the silicon block placement in the epidural space. Rats were returned to their cages and kept on a 37°C heating blanket overnight.

ATK, a preferential and potent inhibitor of cPLA2, was purchased from Cayman Chemicals, MI, USA. Freshly prepared ATK (7.5 mg/kg body weight) with a 0.5% methyl cellulose (Sigma-Aldrich, St Louis, MO, USA) carrier solution was gavage fed to experimental (CEC + ATK) animals 2 h after the CEC and every 24 h thereafter until the end of the experiment. The dose 7.5 mg/kg body weight with carrier methyl cellulose was based on its reported efficacy in a rat model of spinal cord injury [24]. This dose was effective in our studies and was not associated with any adverse effect in the animals. The sham and vehicle (CEC + VEH) groups received only the carrier solution.

### Evaluation of locomotor function

The locomotor function of rats was recorded by rotarod test as previously described from our laboratory [45]. The instrument had a circular column with a diameter of 125 mm. The rats were trained on an automated four-lane rotarod unit supplied by Columbus Instruments (Columbus, OH, USA) for 5 days before surgery. Rotation speed was initially 10 rotations per minute and was increased by five rotations per minute every 5 seconds. The maximum speed was set at 45 rpm. Walking time until the rat fell off the rotating rod was measured five times for each animal at 1 day before surgery and 2, 4, 6, 8, 10, 12, and 14 days after surgery. The inter-experimental gap was 15 min. The mean of five trials was calculated for each rat and represented as mean ± standard deviation (SD).

### Measurement of hyperalgesia and nociception

Animals were acclimatized to the animal chamber of dynamic plantar aesthesiometer (DPA) for about 15 min. DPA is an automated version of von Frey hair analysis (Ugo Basile, Monvalle VA, Italy). It is used to assess changes in sensation or development of mechanical hyperalgesia resulting from neuropathic pain [12]. Animals were placed individually in a small enclosed testing area with a wire mesh floor. The DPA device was positioned beneath the animal so that the filament was directly beneath the plantar surface of the foot to be tested. The instrument raised the filament to touch the foot and progressively increased the force applied until it reached a maximum of 20 g. The force at which the foot was withdrawn was recorded with the software supplied by the manufacturer. Testing was performed once per day until the end of the experiment. Nociception of rats was measured by the paw pressure threshold using analgesy meter (AM) (Ugo Bastille, Monvalle VA, Italy) which applies a linearly increasing mechanical force to the dorsum of the rat’s hind paw. The nociceptive threshold was defined as the force in grams at which the rat withdrew its paw. Basically, the method is similar to one described by Randall and Selitto [46]. Continuously increasing pressure was applied to the dorsal surface of the hind paws. The time the animal withdrew its paw was recorded. Three trials were made on each paw with 5-min inter-test intervals. Testing was performed once per day until the end of the experiment.

### Prostaglandin E2 (PGE2) and leukotriene B4 (LTB4) assay

At the end of the experimental period, the animals were euthanized with an excess dose of Nembutal (Henry Schein, Melville, NY, USA), and 5 to 6 ml of blood was collected by direct cardiac puncture. The serum (50 μl) from each animal was used for PGE2 and LTB4 assays using EIA kits (Cayman, Ann Arbor, MI, USA). Indomethacin (Sigma-Aldrich, St. Louis, MO, USA) at 10 μM was used to inhibit prostaglandin synthase activity immediately after the blood was drawn. The assay system involves the addition of tracer conjugates to the samples. The addition of respective monoclonal antibodies initiated the reaction with bound PGE2/LTB4 conjugate, and the intensity of the reaction was read in a microtiter plate reader at 420 nm. All the samples and the standards were measured in triplicate, and the results are represented as mean ± SD.

### Free fatty acid (FAA) and lysophosphatidylcholine (LPC) determination

Lipids were extracted from the compressed spinal cord regions (epicenter) by the Folch method as described earlier [47,48]. FFA was determined and quantified using high-performance thin layer chromatography (HPTLC) plates [48]. Quantification of LPC was performed by one-dimensional HPTLC (LHPK from Whatman, Inc.; Florham Park, NJ, USA) using the method described by Weerheim *et al*. [49], with modification. Briefly, plates were developed in methyl acetate-1-propanol-chloroform-methyl alcohol-0.25% KCl-acetic acid (100:100:100:40:36.5:2; *v*/*v*/*v*/*v*/*v*/*v*) and visualized by heating at 200°C for 6 min after spraying with 10% CuSO_4_ in 8% phosphoric acid. Different concentrations (0.2 to 5.0 mg) of LPC (1-palmitoyl LPC) were resolved on the same plate as standard for quantification. LPC was quantified by densitometric scanning using the Imaging Calibrated Densitometer (model GS-800; Bio-Rad, Hercules, CA, USA).

### Western blot analysis

Animals from each group were administered lethal doses of Nembutal (150 mg/kg body weight). Compressed spinal cord regions were extracted from the spinal column. Tissue samples were homogenized in a radioimmunoprecipitation assay (RIPA) buffer substituted with protease and a phosphatase inhibitor cocktail (Sigma-Aldrich, St Louis, MO, USA). Homogenate was centrifuged at 13,000 rpm for 20 min at 4°C. The supernatant was collected, and the protein concentrations were determined using a protein assay dye from Bio-Rad Laboratories (Hercules, CA, USA). An equal amount of protein was reduced in an SDS sample buffer (Bio-Rad, Hercules, CA, USA), separated on 4 to 20% Tris–HCl precast gel (Invitrogen, Grand Island, NY, USA) and transferred to a nitrocellulose membrane (Millipore, Billerica, MA, USA). Blocking was done with 5% non-fat milk and incubated overnight at 4°C with primary antibody. The primary antibodies used in the study included phospho-cPLA2 (pcPLA2 Ser^505^), cPLA2, COX-2, p5-LOX, 5-LOX, and mouse anti β-actin monoclonal antibody (1:1000; Sigma, St. Louis, MO, USA). The membranes were briefly washed in tris buffered saline with tween 20 (0.1%) and incubated with respective HRP-conjugated secondary antibody (1:10000; Jackson Laboratory, Bar Harbor, ME, USA) for 1 h at room temperature. Protein bands were visualized using an enhanced chemiluminescence system (Amersham Biosciences, Piscataway, NJ, USA).

### Luxol fast blue (LFB) staining for myelin

Animals were sacrificed with an overdose of Nembutal (150 mg/kg body weight) after 14 days following CEC and perfused transcardially with saline followed by 4% paraformaldehyde (PFA) in 0.1 M sodium phosphate buffer, pH 7.4. The spinal cord and cauda equina fibers were carefully extracted as described [50], and the tissues were post-fixed overnight in PFA. The epicenter and rostral regions from the spinal cord and cauda equina fibers were processed following the histological procedures as previously described from our laboratory [51]. Briefly, the tissues were cut into thin sections of approximately 8-μm thick with rotary microtome (Leica Microsystems, Inc., RM2235, Buffalo Grove, IL, USA). The paraffin sections were deparaffinized with two changes of xylenes and hydrated in 95% ethanol. Slides were left in LFB (Sigma-Aldrich, Cat # L0294, St. Louis, MO, USA) solution (0.1% in 95% ethanol) at 57°C overnight. Excess stain was removed with 95% ethanol and then washed with distilled water. Stained sections were differentiated in lithium carbonate solution (0.05%) for 30 s followed by 70% ethanol for 1 min and washed with distilled water. Sections were counterstained in cresyl violet solution (0.1% in double distilled water) for 1 min. After counterstaining, sections were washed again with distilled water and differentiated in 95% ethanol for 5 min followed by dehydration in 100% ethanol. They were then treated with xylenes each for 5 min and mounted with xylene-based mounting medium. LFB staining intensity was quantified by using Image Pro-Plus 5.1 software (MediaCybernetics, Rockville, MD, USA).

### Statistical analysis

Statistical analysis was performed using GraphPad Prism 5.0 software (GraphPad Software, La Jolla, CA, USA). Statistical significance was determined using paired or unpaired Student’s *t*-test as appropriate, one-way ANOVA with Bonferroni post hoc for three groups, or two-way ANOVA with Bonferroni post hoc for repeated measure analysis. Values were expressed as mean ± standard deviation (SD). *p* values less than 0.05 were considered statistically significant.

## Results

### Cauda equina compression (CEC) model by silicone block implantation

**Figure 1:**
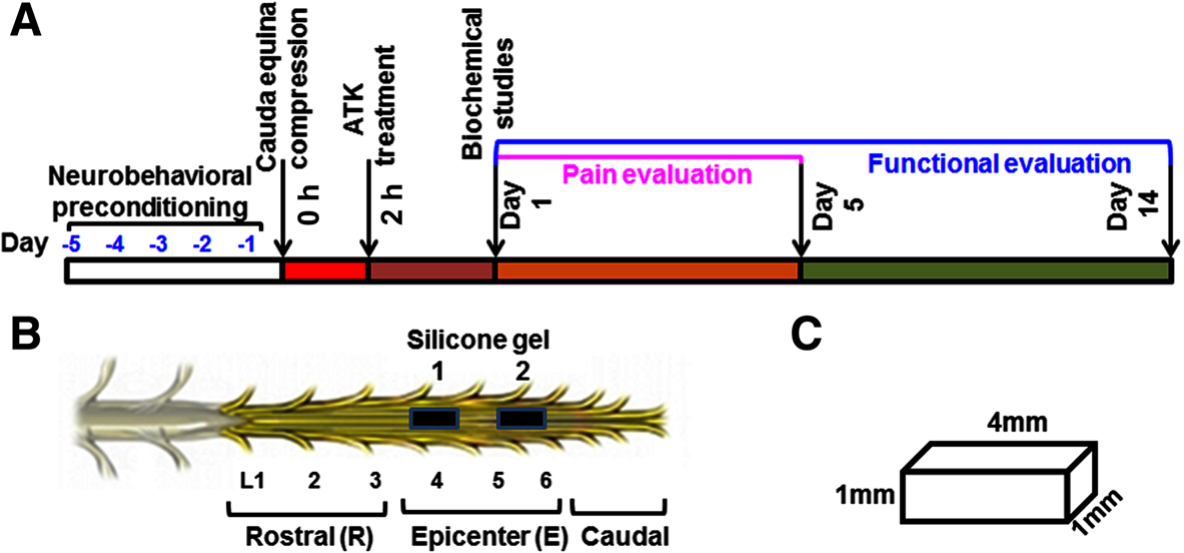
Experimental protocol. Schematic **(A)** shows the timeline of ATK treatment (7.5 mg/kg body weight) at 2 h after CEC begun day 0 and continued for 14 days. Pain was evaluated from days 1 to 5, and motor function was assessed from days 1 to 14 after CEC. Schematic **(B)** shows the location of placement of two silicone gel blocks implanted into the epidural spaces of L4 to L5 and L5 to L6 vertebrae, and schematic **(C)** shows the dimension of silicone gel block.

Two pieces of silicone block (4 mm × 1 mm × 1 mm; Bentec Medical Inc, Woodland, CA, USA) were placed into the epidural spaces between the L4 to L5 and L5 to L6 vertebrae as described in the ‘Materials and methods’ section and depicted in Figure 1. The proper positioning and alignment of the silicone blocks were confirmed by magnetic resonance imaging [51]. This model showed consistent locomotor deficit and hyperalgesia, characteristics of CEC injury, as described from our laboratory [51] and by Watanabe *et al*. [44]. Animals showing hind limb paralysis, insensitivity to tail pinching, and edema at the site of surgery were excluded from the study.

### Effect of CEC on cPLA2 activity and modulation by ATK of cPLA2 and proinflammatory enzymes downstream to cPLA2

**Figure 2:**
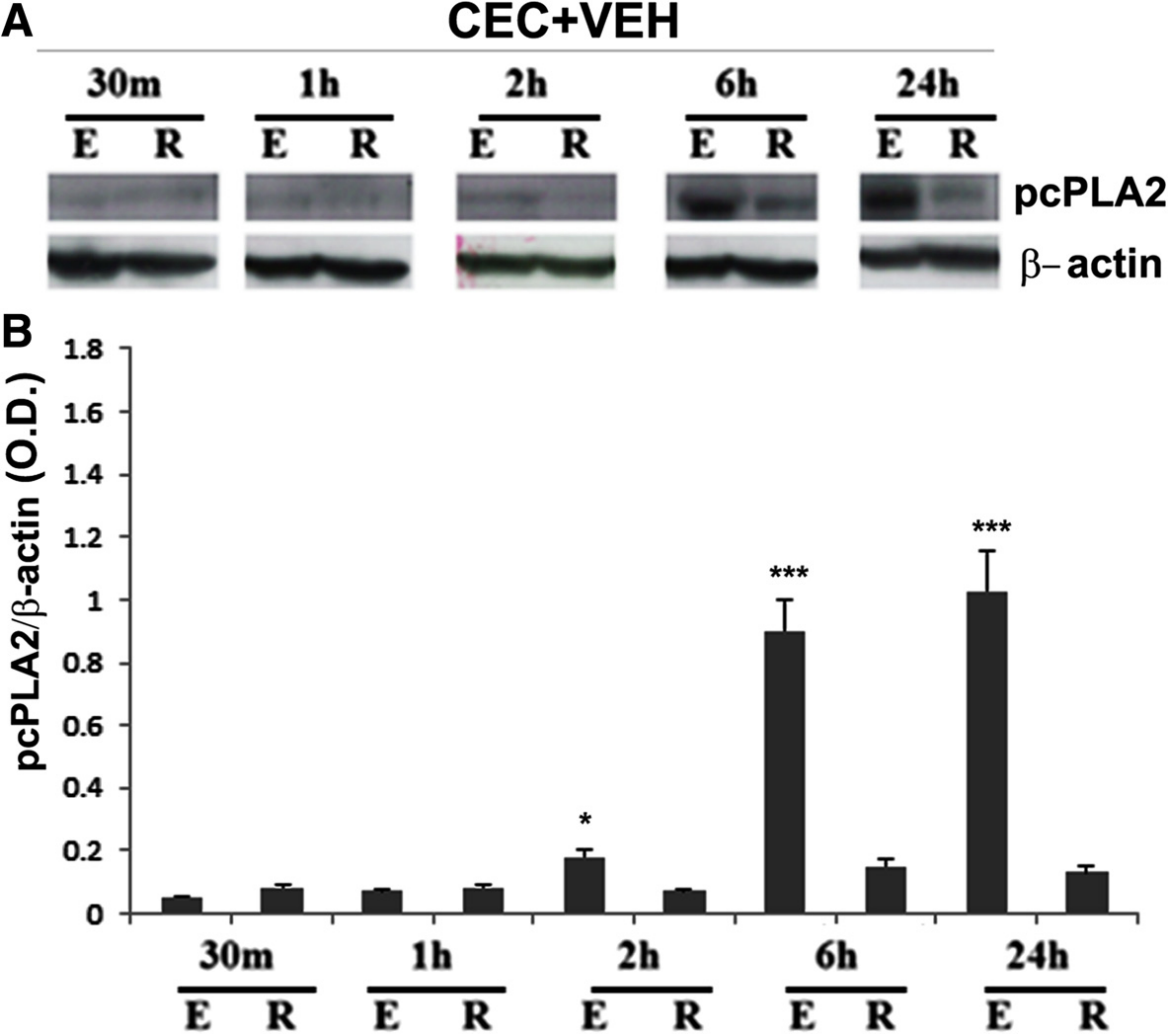
CEC injury activates cPLA2 in spinal cord tissue. Activation of cPLA2 was measured as the levels of phosphorylated cPLA2 (pcPLA2) in spinal cord tissue from epicenter (E) and rostral (R) regions using western analysis **(A)** and its densitometry **(B)**. cPLA2 was activated as early as 2 h after CEC, and it remained significantly activated even at 24 h after CEC. Data are presented as mean ± SD (*n* = 5). *p < 0.05 and ***p < 0.001 *vs*. rostral (R) region of the spinal cord tissue.

**Figure 3:**
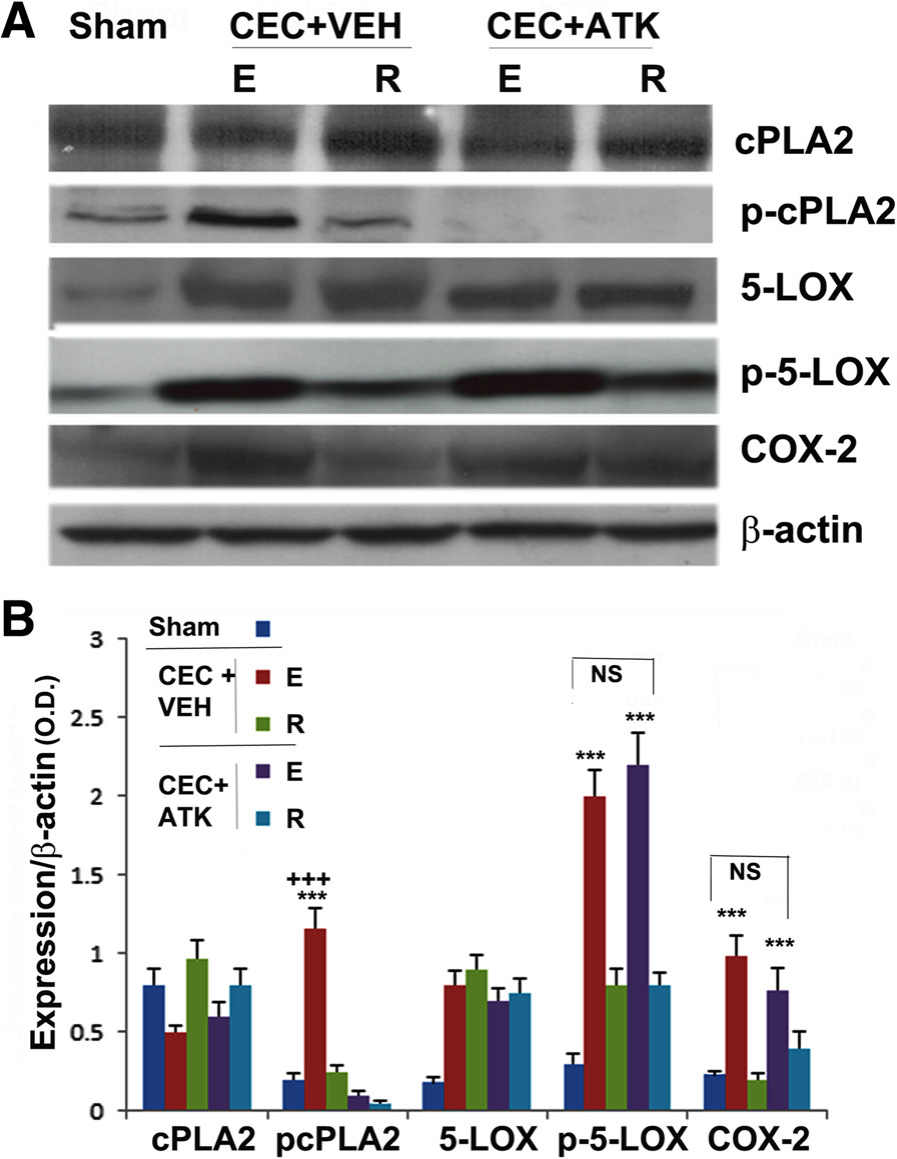
Effect of ATK on CEC-induced increased activity/expression of cPLA2, 5-LOX, and COX-2. Expression of cPLA2, 5-LOX, and COX-2 and activation of cPLA2 and 5-LOX (in terms of increased levels of their phosphorylation) were measured in the spinal cord tissue from epicenter (E) and rostral (R) regions at 24 h after CEC using western analysis **(A)** and its densitometry **(B)**. CEC had no effect on the expression of cPLA2, whereas it increased the expression of p cPLA2, p-5-LOX, 5-LOX, and COX-2 in the epicenter region. While ATK treatment reduced the expression of pcPLA2, the treatment had no significant effects on the expression of p-5-LOX, 5-LOX, and COX-2. Data are presented as mean ± SD (*n* = 5). Statistical significance was evaluated between epicenter (E) and rostral (R) regions in the same group, and E was compared between CEC + VEH and CEC + ATK groups. ***p < 0.001 *vs*. R of the spinal cord tissue from the same group, +++*p* < 0.001 *vs*. E of the spinal cord from CEC + ATK.

Using Western blot analysis, we determined the effect of CEC on cPLA2 activation (phospho-cPLA2 or pcPLA2) from 30 min to 24 h and the effect of ATK on the expression of cPLA2 and its downstream signaling mediators (5-LOX and COX-2) at 24 h. We found a significant and sustained activation of cPLA2 in terms of increased levels of pcPLA2 from 2 h onward in the epicenter (E) of the CEC spinal cord (Figure 2A, B; western and densitometry, respectively). The expression of cPLA2 protein 24 h after injury did not change significantly in CEC + VEH *vs*. CEC + ATK spinal cord tissue. However, ATK treatment of CEC rats significantly decreased the level of pcPLA2 in the epicenter of spinal cord tissue at 24 h following CEC (Figure 3A, B; western and densitometry, respectively). In contrast, ATK treatment neither reduced the levels of p-5-LOX nor decreased the expression of 5-LOX and COX-2 in the very same spinal tissue (Figure 3A, B; western and densitometry).

### ATK treatment of CEC decreased the levels of FFA, LPC, PGE2, and LTB4

**Figure 4:**
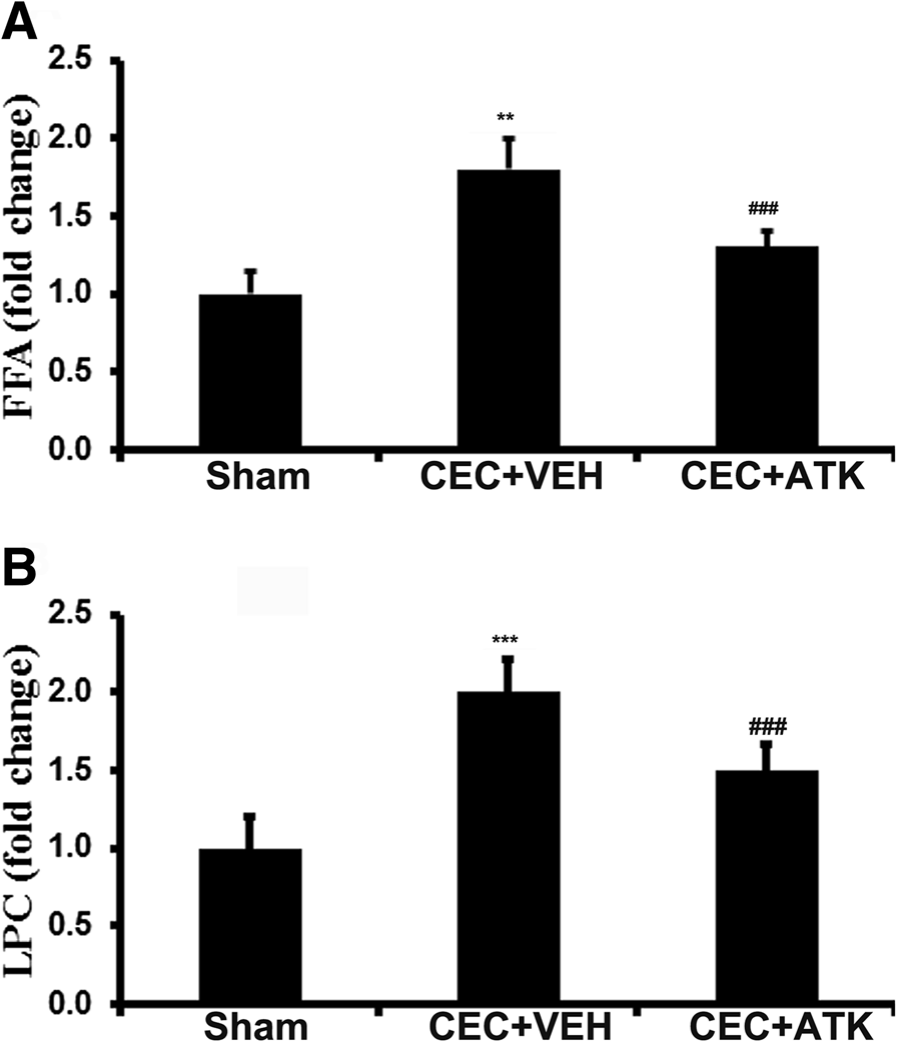
Effect of ATK on CEC-induced increased levels of FFA and LPC in the spinal cord tissue from epicenter region 24 h after CEC. Levels of FFA **(A)** and LPC **(B)** were measured by HPTLC. Data are expressed as means ± SD (*n* = 5). **p < 0.01, ***p < 0.001 *vs*. Sham and CEC + ATK, ###*p* < 0.001 *vs*. Sham.

To test whether cPLA2 activation was responsible for increased levels of phospholipid degradation products and whether ATK decreased these levels, we analyzed the content of FFA and LPC in the epicenter of the spinal tissue. We found that the levels of both FFA (Figure 4A, *p* < 0.01) and LPC (Figure 4B, *p* < 0.001) were significantly increased in the CEC + VEH group. The ATK treatment significantly reduced CEC-mediated increased levels of FFA (measured by HPTLC) and LPC (measured by HPTLC) in the epicenter of the spinal tissue (Figure 4A, B). However, the levels of both FFA and LPC never normalized to the sham levels in the CEC + ATK group (Figure 4A, B). We did not measure the levels of free AA because the implicated cPLA2 is reported to hydrolyze mainly AA from the *sn*-2 position of phosphatidylcholine [38].

**Figure 5:**
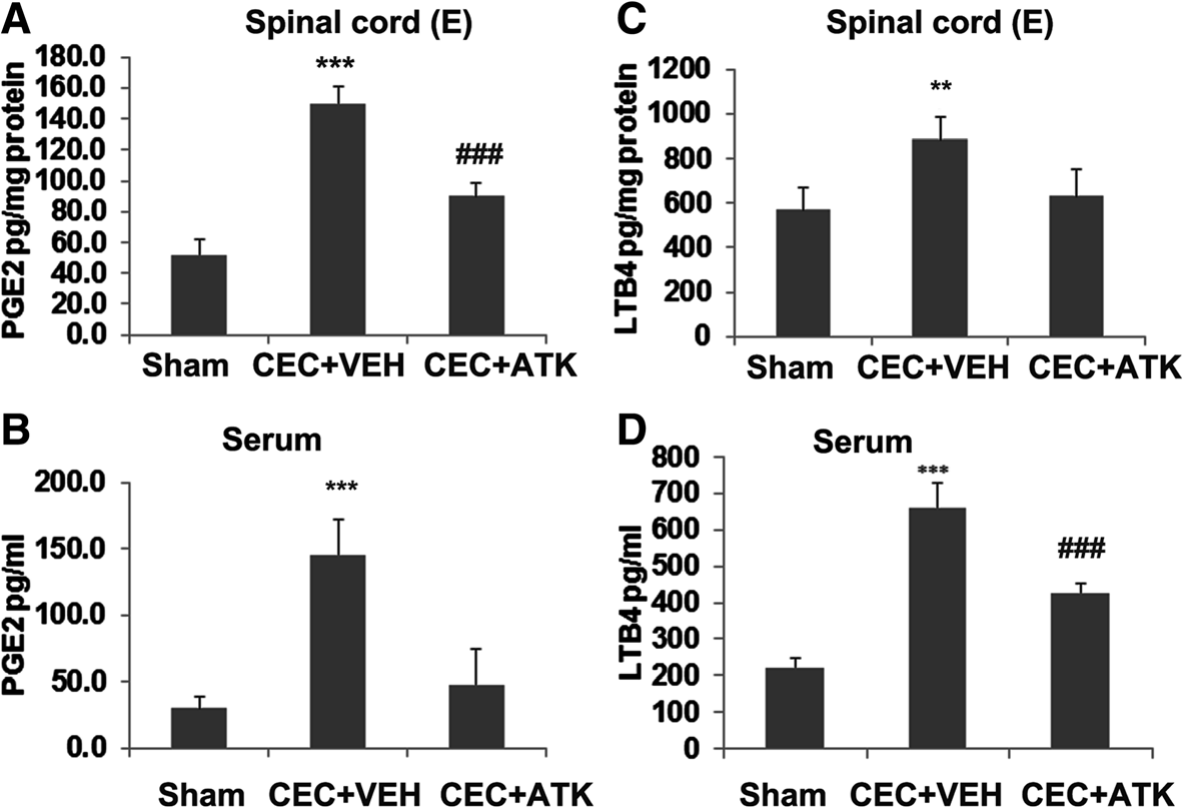
Effect of ATK on CEC-induced increased levels of PGE2 and LTB4. The levels of PGE2 **(A, B)** and LTB4 **(C, D)** were measured using ELISA at 24 h after CEC in the spinal cord tissue from epicenter region (E) and serum. The levels of both PGE2 and LTB4 were significantly increased in CEC + VEH group in the spinal cord (A, C) as well as in the serum (B, D). ATK treatment decreased the levels of both. Data are expressed as mean ± SD (*n* = 5). Results are presented as pg/mg protein for spinal cord tissue and pg/ml in serum. **p < 0.01, ***p < 0.001 *vs*. Sham and CEC + ATK, ###*p* < 0.001 *vs*. Sham.

Free AA is metabolized mainly into proinflammatory eicosanoids including PGE2 (a product of COX-2) and LTB4 (a product of 5-LOX). Both PGE2 and LTB4 levels were measured in both the spinal cord tissue and serum from CEC + VEH and CEC + ATK animals after 24 h (Figure 5). CEC + VEH animals had significantly higher levels of PGE2 (*p* < 0.001) than sham animals at 24 h after CEC in both the spinal cord (Figure 5A) and serum (Figure 5B). Similarly, the levels of LTB4 were significantly higher than sham animals in the spinal cord (Figure 5C, *p* < 0.01) and serum (Figure 5D, *p* < 0.001) from CEC + VEH animals. ATK treatment of CEC significantly reduced the levels of both PGE2 and LTB4 in the spinal cord tissue as well as in the serum (Figure 5). However, PGE2 levels in spinal cord and LTB4 levels in serum never normalized to sham levels.

### ATK treatment of CEC protects against loss of myelin in spinal tissue and cauda equina fibers

**Figure 6:**
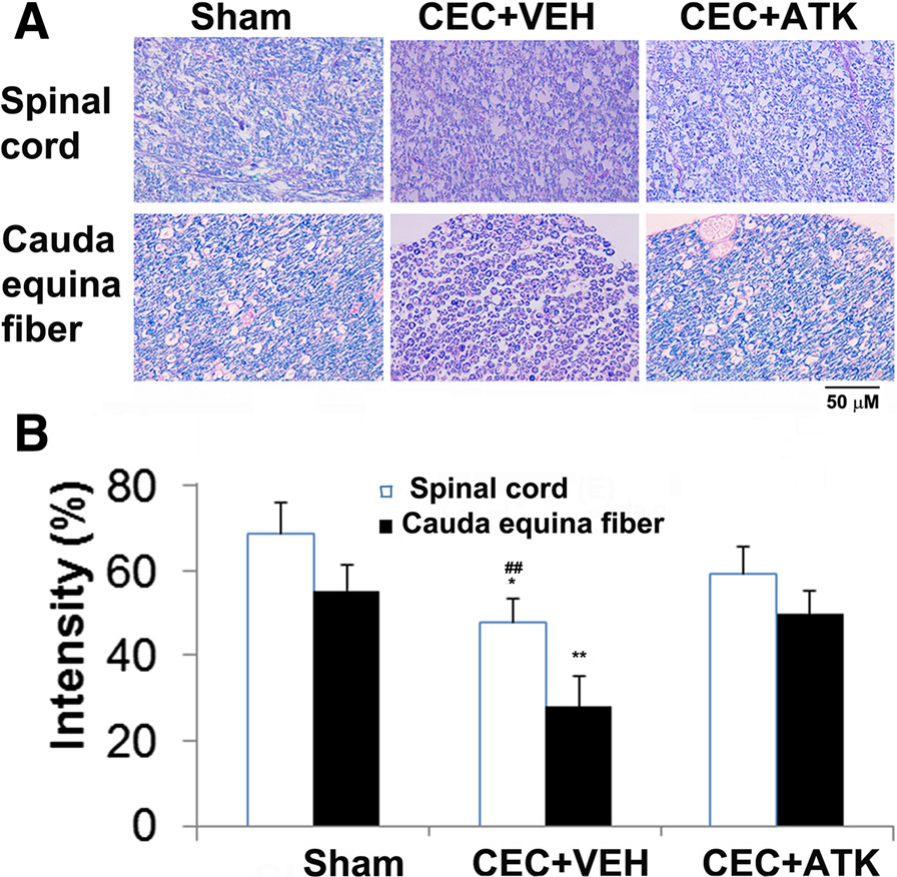
Effect of ATK on CEC-induced changes in LFB histochemistry of the spinal cord tissue from epicenter region (upper panel) and CE fibers (lower panel) 14 days after CEC. Myelin levels in both the spinal cord and CE fibers were significantly reduced in CEC + VEH group compared to sham and CEC + ATK groups **(A)**. Corresponding quantification of LFB staining intensity was given in **(B)**. Photomicrographs are representative of *n* = 5 in each group. *p < 0.05 *vs*. CEC + ATK, ##*p* < 0.01 *vs*. Sham, **p < 0.01 *vs*. Sham and CEC + ATK.

Spinal cord sections proximal to the CEC region and CE fibers were stained with LFB for myelin on day 14 following CEC (Figure 6). Spinal cord from the CEC + VEH group showed a significant reduction in the levels of myelin (Figure 6A, B). The LFB staining in the CEC + ATK group was similar to the sham group. A significantly similar trend for the LFB staining was observed in CE fibers. While fibers from the CEC + VEH group showed significantly reduced myelin staining, the CEC + ATK group had LFB staining similar to the LFB staining of the sham group (Figure 6A, B).

### ATK treatment of CEC enhanced the pain threshold

**Figure 7:**
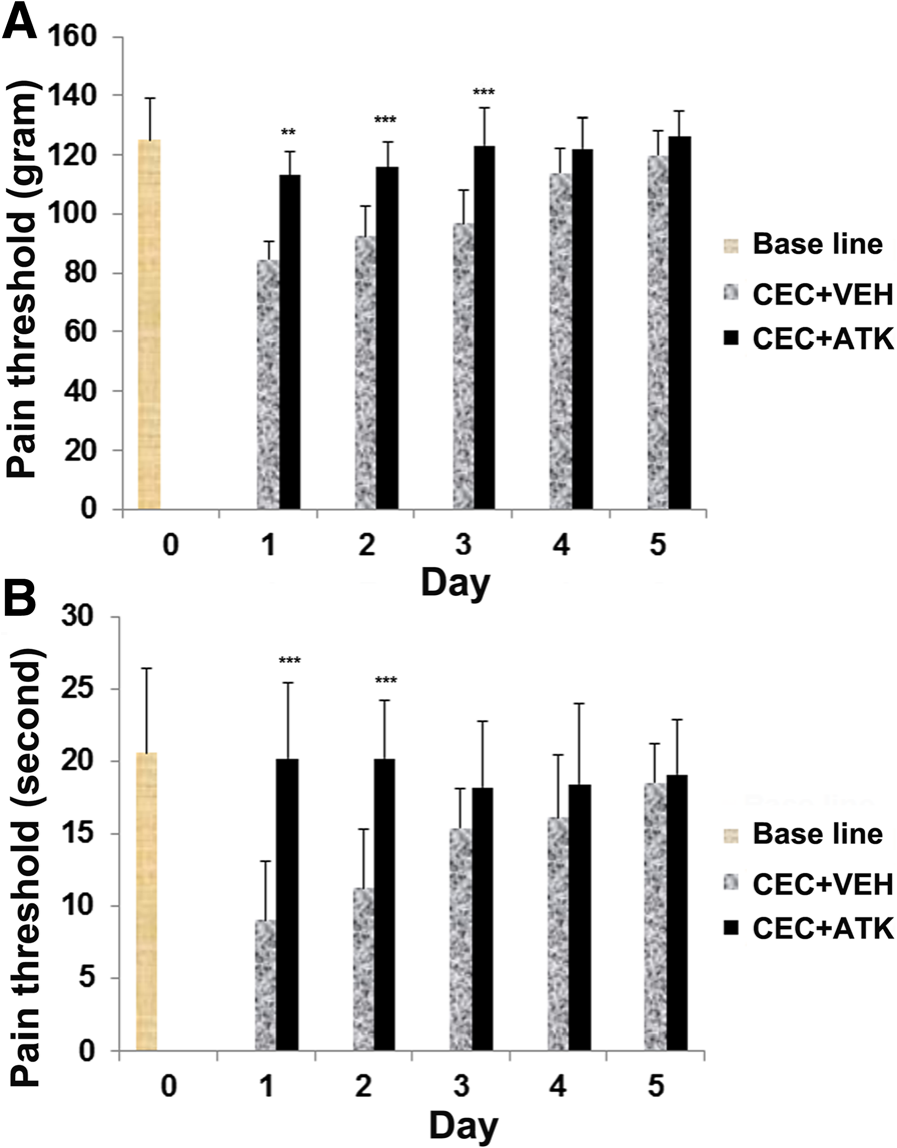
Effect of ATK on CEC-decreased threshold of pain. Pain threshold in animals was measured with AM **(A)** and DPA **(B)** for 5 days following CEC. Baseline represents the pain threshold of rats before CEC. CEC + VEH group was hyperalgesic when compared to respective ATK-treated group at least for 3 days following CEC. ATK treatment significantly decreased the hyperalgesia associated with CEC. Data are presented as mean ± SD (*n* = 8). **p < 0.01, ***p < 0.001 *vs*. CEC + VEH.

Pain threshold was determined in both the CEC + VEH and the CEC + ATK groups and compared with baseline from the same animals. The pain threshold of rats was measured from day 0 to 5 using AM (Figure 7A) and DPA (Figure 7B). We did not measure pain threshold after 5 days of CEC because we could not find significant hyperalgesic differences 6 days after CEC when compared to baseline. The baseline pain threshold of rats was measured on day 0 (day before surgery). ATK treatment increased the pain threshold of the injured animals. On days 1 and 2 following the treatment, the difference in pain threshold between the CEC + VEH and CEC + ATK animals was significant. CEC + VEH animals were significantly sensitive to hyperalgesia and allodynia. However, CEC + ATK animals had thresholds comparable to baseline values, indicating that ATK decreased CEC-induced pain.

### ATK treatment of CEC improved locomotor function evaluated by rotarod task

**Figure 8:**
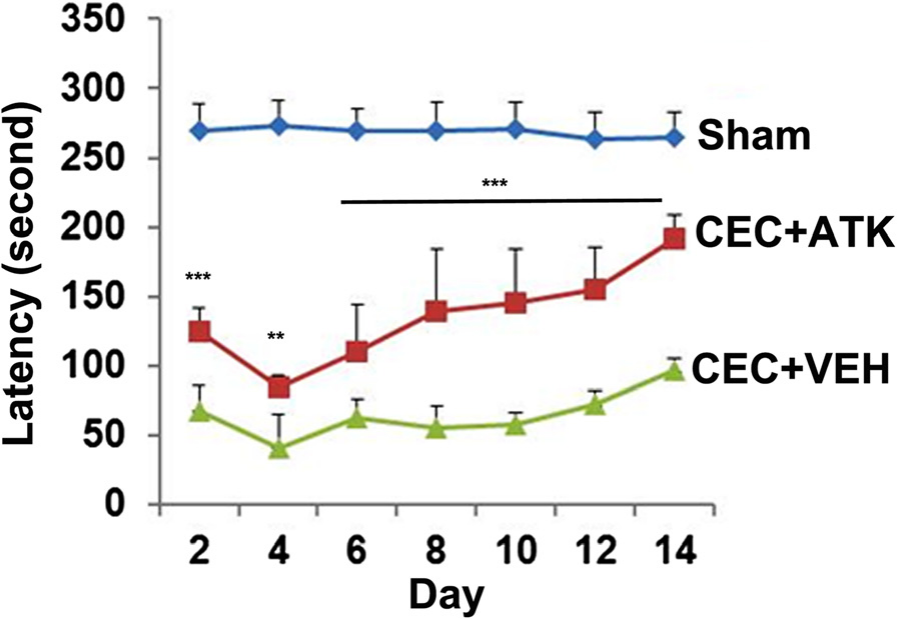
Effect of ATK on CEC-induced locomotor deficits. Animals were treated with ATK orally at 2 h following CEC, and the same dose was repeated daily until the end point (14 days). Motor function was evaluated by rotarod task on indicted days. Sham-operated animal had normal motor function (latency 269 ± 19 s). Data are presented as mean ± SD (*n* = 8). **p < 0.01, ***p < 0.001 *vs*. CEC + VEH. Neither group reached sham levels over the testing period (p < 0.001).

Inhibition of cPLA2 using ATK has been previously shown to improve locomotor function in a rat model of contusion spinal cord injury [24]. Therefore, we anticipated that ATK would also be effective in our LSS model in restoring motor function. Animals were pre-trained on the rotarod for 5 days before the experiments began (Figure 1). All animals were able to walk on rotarod for 250 ± 18 s 1 day before the injury. Animals in each group were tested on rotarod on alternate day until 14 days after the surgery (Figure 8). The CEC + VEH group showed a severe motor deficit following CEC. On day 2 post injury, the CEC + VEH group had a latency of 68 ± 17 s. With the progression of natural healing, the walking efficiency increased to 97 ± 8 s on the 14th day. The CEC + ATK group started showing significant improvement (*p* < 0.001) in motor function from the 2nd day (140 ± 45 s) to the endpoint (14th day; 191 ± 8 s) (Figure 8). Sham-operated animals did not show any significant change in the latency on any day tested.

## Discussion

**Figure 9:**
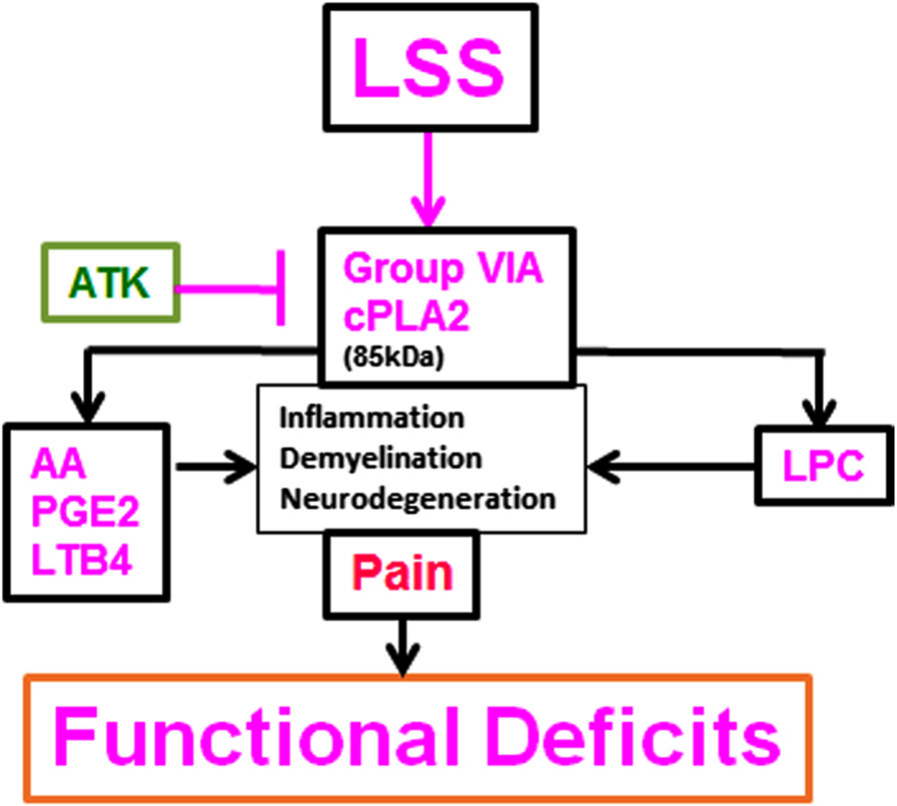
Schematic showing the hypothesized LSS-mediated events leading to activation of cPLA2, production of injurious lipids and induction of inflammation pain and motor function deficit. ATK treatment of CEC ameliorates LSS injury by inhibiting the activity of cPLA2.

Motor function deficits and neuropathic pain are the hallmarks of LSS and the associated LBP [52]. The current study provides pharmacological evidence for the therapeutic potential of the cPLA2 inhibitor ATK in aiding functional recovery and reducing pain in a rat model of LSS. Our findings demonstrate for the first time that the administration of ATK in a CEC animal model of LSS significantly restored locomotor function and increased the pain threshold of the hyperalgesic CEC rats. The beneficial effects of ATK correlated well with both the inhibition of cPLA2 activation and the reduction of cPLA2-derived proinflammatory and nociceptive lipid mediators LPC, PGE2, and LTB4 as depicted in the schematic (Figure 9).

The lack of satisfactory treatment of LSS is partially the result of limited understanding of the mechanisms of neuroinflammatory injury and neuropathic pain. Neuropathic pain is characterized by sensory abnormalities, such as abnormal sensation (dysestehesia), increased response to painful stimuli (hyperalgesia), and response to a stimulus that does not usually induce pain (allodynia) [53]. Following spinal trauma or constriction, cPLA2 is an important contributor to both neuroinflammation and neuropathic pain [24,54,55]. Activated spinal neurons, astrocytes and microglia are involved in cPLA2-induced SCI. cPLA2-derived bioactive lipid mediators, including LPC and eicosanoids such as PGE2 and leukotrienes, are causally implicated in animal models of SCI and spinal nociceptive processing [13,24,55]. cPLA2 reduction has been shown to improve functional recovery in stroke [56], Alzheimer [57], and multiple sclerosis [42], indicating its causative role in neurodegeneration. To the best of our knowledge, the role of cPLA2 in LSS or CEC is not known. The cPLA2 message is predominant in rat spinal cord [55], expressed in the neurons and oligodendrocytes of contused spinal cord [13], suggesting that upregulation of this enzyme after trauma may be responsible for the secondary injury, thus the inflammation, nociception, and functional deficits following LSS. In this study, CEC significantly activated cPLA2 (measured as pcPLA2) in the epicenter region, which was observed as early as 2 h following injury, and the expression of pcPLA2 remained high and sustained even 24 h after injury (Figure 2). To reduce the activation of cPLA2, we treated CEC animals with ATK 2 h after the injury and observed that ATK reduced the levels of pcPLA2 in the epicenter region when measured 24 h post injury (Figure 3). However, ATK had no effect on the expression of cPLA2 (Figure 3). The activity of cPLA2 has been shown to be effectively inhibited by ATK, and ATK treatment of contusive SCI confers anti-inflammatory and neuroprotective effects, leading to reduced hyperalgesia and increased functional recovery [13,55]. Although ATK also inhibits the activity of iPLA2 to a limited extent, its role in SCI has been reported to be less significant [55]. The iPLA2 inhibitor bromoenol lactone had no beneficial effects as observed with ATK following SCI [55], supporting this less significant role of iPLA2 in SCI. The anti-inflammatory effect of ATK has also been reported via the inhibition of the activity, and not the expression, of COX-1 and COX-2 in cell culture studies [58]. However, the direct effect of ATK on stenosis is not known, and our studies did not evaluate its anti-stenotic or anti-ischemic effect.

Prominent enzymes downstream to cPLA2, 5-LOX, and COX-2 produce proinflammatory leukotrienes, such as LTB4, and prostaglandins, such as PGE2. COX-2 is inducible, whereas 5-LOX is activated after SCI. Therefore, we determined the effect of CEC and ATK on the expression of 5-LOX and COX-2 and the levels of p-5-LOX 24 h following injury onset (Figure 3). ATK treatment neither reduced CEC-mediated significantly increased expression of 5-LOX and COX-2 nor did it reduce the remarkably high levels of p-5-LOX in the CEC spinal cord, indicating that ATK mediated its effect in this model by inhibiting cPLA2. An indication of the effective inhibition of cPLA2 was shown by reduced levels of LPC and FFA, products of PC hydrolysis by cPLA2, in CEC + ATK (Figure 4). LPC and FFA had similar patterns of results, suggesting that both may have originated from PC by the action of cPLA2. In this study, we did not measure the levels of free AA because PC is known to have mainly AA at its *sn*-2 position. Therefore, FFA represents mainly free AA. Free AA is metabolized to prostaglandins by COX-2 and leukotrienes by 5-LOX. We measured PGE2 as representative of COX-2 products and LTB4 as representative of 5-LOX products. The levels of both were significantly increased in the epicenter region of the spinal tissue as well as in the serum (Figure 5). PGE2 has been implicated in inflammation and neuropathic pain following SCI [26,32,59]. Similar to PGE2, LTB4 has been shown to accumulate following spinal cord injury and contribute to neuropathic pain [60,61]. ATK treatment of CEC significantly decreased the levels of both PGE2 and LTB4 (Figure 5), showing that they originated from cPLA2. PGE2 production can also be blocked by inhibition of COX-2; however, COX-2 inhibition is associated with undesirable vascular effects [62,63]. Also, the advantage associated with the inhibition of cPLA2 is that it blocks the production of not only eicosanoids but also LPC.

LPC is a signaling molecule involved in chronic inflammation and tissue damage [64]. It is held responsible, at least in part, for several deleterious effects of cPLA2 in brain, such as demyelination and axonal degeneration [65]. The pain stimulatory nociceptive effects of LPC are mainly mediated by its hydrolyzed product lysophosphatidic acid (LPA) [66,67]. LPC is converted to LPA by autotaxin, and LPA mediates its effect through at least six different receptors [66]. Blocking cPLA2 by ATK has been shown to reduce LPA receptor activity and nerve injury-induced neuropathic pain [68]. In our studies, ATK treatment of CEC decreased the levels of LPC (Figure 4) as well as pain (Figure 7), indicating that cPLA2 inhibition is an ideal strategy to reduce hyperalgesia. LPC-induced demyelination following nerve injury has also been shown through the autotaxin/LPA mechanism [69], indicating again the involvement of cPLA2 activity. Myelin loss is an intrinsic component of CEC injury as observed (Figure 6) and previously reported from our laboratory [51,70]. Reduced loss of myelin in both the spinal cord and cauda equina fibers after CEC in ATK-treated animals (Figure 6) supports the involvement of the possible cPLA2/LPC/LPA pathway in CEC-induced demyelination.

Neurological deficits (walking disability associated with motor function deficits) are a characteristic of LSS [2] and the CEC animal model [71]. Motor function recovery is a major objective of LSS basic research to determine the therapeutic efficacy of a drug. A significant motor function walking deficit was observed from 2 days onward in this study (Figure 8), validating the CEC model. Similar functional deficits have previously been reported in this model [44,51]. cPLA2 activity has been shown to compromise motor functions in SCI animal models [13]; therefore, our observation of improved walking ability in the CEC + ATK group supports that cPLA2 is a causative factor in CEC-induced motor function deficits.

## Conclusion

In the present study, we have shown the therapeutic potential of the cPLA2 inhibitor ATK in a CEC animal model of LSS. The observed beneficial effect may be primarily due to the inhibition of cPLA2 and the reduction of proinflammatory lipid mediators originating from the CEC-induced activation of cPLA2. Because clinically proven LSS therapy is not currently available and ATK confers clinically relevant beneficial effects in our rat model of CEC, further investigation is warranted to test ATK’s efficacy in human LSS.

## Abbreviations

AA: arachidonic acid
AM: analgesy meter
ATK: arachidonyl trifluoromethyl ketone
CEC: cauda equina compression
COX: cyclooxygenase
DPA: dynamic plantar aesthesiometer
cPLA2: cytosolic phospholipases A2
FFA: free fatty acids
HPTLC: high-performance thin layer chromatography
LBP: low back pain
LFB: luxol fast blue
LOX: lipoxygenase
LPA: lysophosphatidic acid
LPC: lysophosphatidylcholine
LSS: lumbar spinal stenosis
LTB4: leukotriene B4
NO: nitric oxide
PC: phosphatidylcholine
PGE2: prostaglandin E2
SD: standard deviation
VEH: vehicle
